# A prediction and imputation method for marine animal movement data

**DOI:** 10.7717/peerj-cs.656

**Published:** 2021-08-03

**Authors:** Xinqing Li, Tanguy Tresor Sindihebura, Lei Zhou, Carlos M. Duarte, Daniel P. Costa, Mark A. Hindell, Clive McMahon, Mônica M.C. Muelbert, Xiangliang Zhang, Chengbin Peng

**Affiliations:** 1 College of Information Science and Engineering, Ningbo University, Ningbo, China; 2 Red Sea Research Center, King Abdullah University of Science & Technology, Thuwal, Saudi Arabia; 3 Department of Ecology & Evolutionary Biology, University of California, Santa Cruz, Santa Cruz, United States of America; 4 Institute for Marine and Antarctic Studies, University of Tasmania, Tasmania, Australia; 5 Sydney Institute of Marine Science, Mosman, Australia; 6 Instituto de Oceanografia, Rio Grande, Brazil; 7 Computer, Electrical and Mathematical Sciences and Engineering, King Abdullah University of Science & Technology, Thuwal, Saudi Arabia; 8 Ningbo Institute of Industrial Technology, Chinese Academy of Sciences, Ningbo, Zhejiang, China

**Keywords:** Marine animal movement, Trajectory analysis, Prediction, Imputation

## Abstract

Data prediction and imputation are important parts of marine animal movement trajectory analysis as they can help researchers understand animal movement patterns and address missing data issues. Compared with traditional methods, deep learning methods can usually provide enhanced pattern extraction capabilities, but their applications in marine data analysis are still limited. In this research, we propose a composite deep learning model to improve the accuracy of marine animal trajectory prediction and imputation. The model extracts patterns from the trajectories with an encoder network and reconstructs the trajectories using these patterns with a decoder network. We use attention mechanisms to highlight certain extracted patterns as well for the decoder. We also feed these patterns into a second decoder for prediction and imputation. Therefore, our approach is a coupling of unsupervised learning with the encoder and the first decoder and supervised learning with the encoder and the second decoder. Experimental results demonstrate that our approach can reduce errors by at least 10% on average comparing with other methods.

## Introduction

With the advancement in tracking devices, vast amounts of trajectory data have been collected. As a consequence, research in trajectory data prediction, clustering, and imputation is proliferating. The latest developments in position tracking and data analysis techniques have dramatically changed the way researchers study wildlife movements. Interdisciplinary collaborations have led to the development of new quantitative methods and tools that have become key to animal movement research and allow for enhanced and extensive interpretation of the results (Jonsen, Flemming & Myers, 2005; Johnson et al., 2008; MA et al., 2020). Because animals obtain resources such as prey and mates through movements, their movement patterns can contain essential biological information. Thus, researchers analyzing animal data obtained from remote sensing technology can help them determine places that animals like, understand their migration strategies, and enhance the effectiveness of protecting endangered species (Calenge, Dray & Royer-Carenzi, 2009).

Recent research has shown that marine animals vary significantly in their movement patterns in response to various physical and biological factors. For example, by investigating a multi-year database of female southern elephant seal motion behaviors, some studies have shown that the preference of female seals for middle scale ocean circulation is seasonally flexible (Cotté et al., 2015). Statistical data analysis has also revealed a link between elephant seal behavior and ocean patterns and suggested that pre-reproductive female southern elephant seals prefer to forage near mesoscale fronts (Campagna et al., 2006). From these examples, we can realize that a time varying trajectory analysis model is crucial because it can reveal unknown information from ecological data and provide models for observations. One simple way to achieve this is to allow the model output depending on the input values from previous inputs, and some deep learning approaches can be used.

Deep learning methods have been successfully used in many applications. In image classification and object detection, methods based on deep convolutional neural networks can achieve excellent results (Perez & Wang, 2017; Zhao et al., 2019). In time series analysis, methods based on recurrent neural networks perform well (Connor, Martin & Atlas, 1994). Researchers have also found that recurrent neural networks have an advantage over feedforward neural networks over time series and get better results on electric load forecasting (Connor, Martin & Atlas, 1994). To extract patterns in an unsupervised way, researchers have proposed auto-encoders to reconstruct input data and to learn patterns simultaneously (Vincent et al., 2008).

However, most trajectory analysis research using deep learning tools usually focuses on human trajectories (Ma et al., 2019; Rudenko et al., 2020), which are quite regular on a daily basis. As marine animal trajectories can have very different patterns, many existing approaches are not applicable. In this work, we propose to model marine animal trajectories based on encoding and decoding modules for prediction and imputation. Our contributions are as follows:

First, we propose a deep learning-based approach for marine animal trajectory data analysis, specifically, prediction and imputation within the same framework.

Second, we design a learning model integrating recurrent neural networks and auto-encoder networks along with attention modules to model marine animal trajectory data with better accuracy.

Third, our model utilizes hidden patterns of trajectories from encoders to improve prediction and imputation accuracy.

The remaining parts are organized as follows. In ‘Related works’, we state the interaction between trajectory and environment and the superiority of recurrent neural networks in dealing with time series problems. In ‘Method’, we described our model in detail and explained how the data is transformed in our model. In ‘Experiments’, we compare our model with other algorithms and preprocess the data in two different ways to demonstrate our method’s performance and efficiency. We conclude this work in ‘Conclusion’.

## Related Works

Animal trajectories are generally affected by animal behaviors as well as situational and environmental factors. Therefore, it is not suitable to describe these trajectories with specific distributions, and flexible non-linear models are more preferable to identify underlying patterns.

Many machine learning methods have been used to analyze movement data for cows (Martiskainen et al., 2009), cheetahs (Grünewälder et al., 2012), penguins (Carroll et al., 2014), etc. For example, random forest is widely used for movement data prediction or imputation (Zhang et al., 2020; Lin et al., 2017; He et al., 2019). State-space models (Breed et al., 2012), hidden Markov models (Michelot, Langrock & Patterson, 2016), and Gaussian mixture models have also been used extensively in identifying and modeling telemetry data (Gibb et al., 2017; Jonsen et al., 2018; Langrock et al., 2012). Across many of these cases, particular patterns have usually been manually extracted from the data to simplify the predictive task (Jonsen et al., 2018).

Artificial neural networks are another kind of feasible methods. Such models have been used to estimate the movement probability of elks by considering the physical spatial structure of landscapes and animal memory of previously visited locations (Dalziel, Morales & Fryxell, 2008). Artificial neural networks can also identify and predict diving activities of seabirds (Browning et al., 2018). If inputs are sequences, a special type of artificial neural networks, recurrent neural networks can be used as they can learn the implicit temporal dependencies in sequential or spatial–temporal data. They have shown obvious advantages in dealing with problems such as time series prediction (Connor & Atlas, 1991), speech recognition (Graves, Mohamed & Hinton, 2013), subtitle generation (Song et al., 2019), image or video classification (Yang, Krompass & Tresp, 2017), handwriting sequences (Graves, 2013). Recurrent neural networks can also predict image sequences, and it performs well in action recognition when combining with auto-encoders (Srivastava, Mansimov & Salakhudinov, 2015). They can also be used for machine translation when using two-way recurrent neural networks (Cho et al., 2014; Graves & Jaitly, 2014). Some studies have also used recurrent neural networks with random forest interpolation for pattern refinement to improve the prediction performance of recurrent neural networks (Rew et al., 2019).

To further improve the prediction and imputation performance, in this work, we propose to use an encoder and one decoder for trajectory embedding and use the other decoder for trajectory prediction and imputation. Experimental results justify the effectiveness.

## Method

### Movement analysis framework

**Figure 1:**
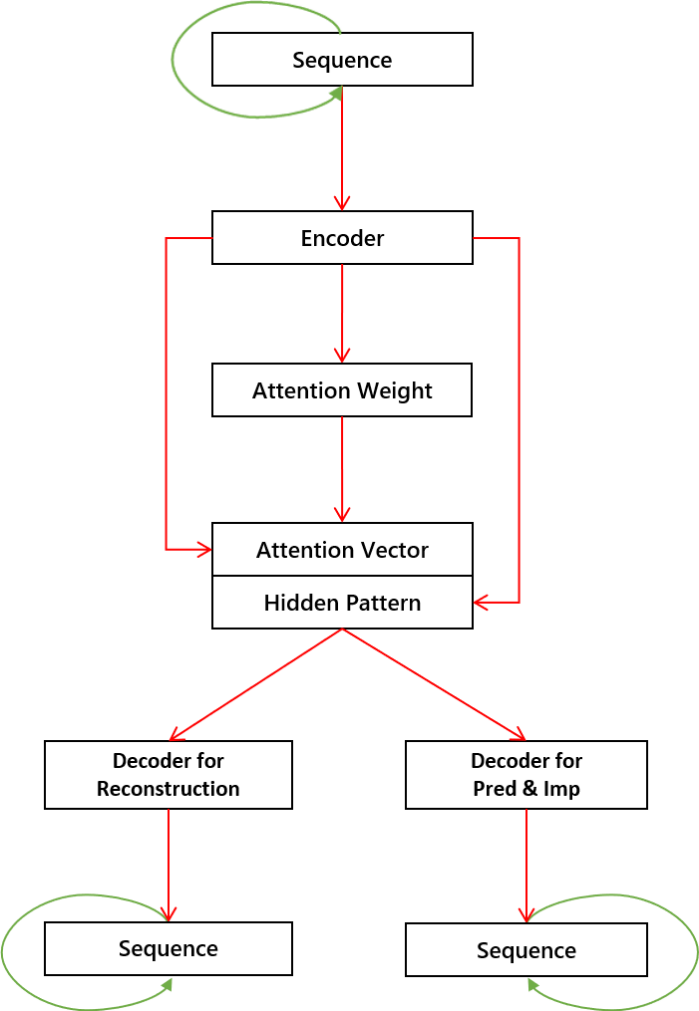
The proposed model with one encoder, one attention module, and two decoders. Each green line indicates that its connected module is recursive. The concatenation of attention vectors and hidden patterns serves as input for decoders.

Auto-encoders are usually used for unsupervised learning, which requires unlabeled data only. In this work, we propose a novel framework that integrates auto-encoders, recurrent neural networks, and attention modules, to improve the prediction and imputation performance for marine animal trajectories. The proposed framework differs from traditional approaches as it has an attention module for the encoder output, and it has two decoders for two purposes, as shown in Fig. 1. The first decoder can reconstruct input data and learn patterns through the reconstruction process, while the second one can perform trajectory prediction and imputation from learned patterns.

#### LSTM encoder

Long-short-term-memory (LSTM) network is a kind of recurrent neural network. LSTM network is suitable for processing and predicting events for relatively long intervals in time series. At the same time, in terms of performance, LSTM networks are usually superior to ordinary recurrent neural networks (Gers, Schmidhuber & Cummins, 1999). Here, we briefly describe the basic building block, an LSTM cell (Graves, 2013). An LSTM cell differs from a typical recurrent neural cell in that it controls the flow of information through input gates, forget gates, and output gates.

In this part, we use a *T* × *F* matrix *x* to represent an input trajectory with *T* time steps and *F* features. We use a row vector *x*_*t*_ represent the trajectory features at time step *t*. Similarly, we use *h*, *f*, *i*, and *o* to represent the hidden states, forget states, input states, and output states respectively, and we use subscript *t* to represent these values at time step *t*. In an LSTM cell for time step *t*, *h*_*t*−1_ and *x*_*t*_ can be used to calculate forget state *f*_*t*_, input state *i*_*t*_, output state *o*_*t*_, and candidate cell state ${\widetilde {C}}_{t}$, as represented from Eq. (1) to Eq. (4). In these equations, *W*_*f*_, *W*_*i*_, *W*_*o*_, and *W*_*C*_ are weight matrices. Function *σ* is a softmax activation function and *tahn* is a tahn activation function. Then, we combine the previous cell state *C*_*t*−1_ and the candidate cell state ${\widetilde {C}}_{t}$ weighted by forget state and input state respectively, as shown in Eq. (5). Hidden state *h*_*t*_ is updated with output state *c*_*t*_ and current cell state *C*_*t*_ as shown in Eq. (6).

(1) \begin{eqnarray*}& & {f}_{t}=\sigma ({W}_{f}\cdot [{h}_{t-1},{x}_{t}]+{b}_{f}),\end{eqnarray*}

(2) \begin{eqnarray*}& & {i}_{t}=\sigma ({W}_{i}\cdot [{h}_{t-1},{x}_{t}]+{b}_{i}),\end{eqnarray*}

(3) \begin{eqnarray*}& & {o}_{t}=\sigma ({W}_{o}\cdot [{h}_{t-1},{x}_{t}]+{b}_{o}),\end{eqnarray*}

(4) \begin{eqnarray*}& & {\widetilde {C}}_{t}=tanh({W}_{C}\cdot [{h}_{t-1},{x}_{t}]+{b}_{C}),\end{eqnarray*}

(5) \begin{eqnarray*}& & {C}_{t}={f}_{t}\ast {C}_{t-1}+{i}_{t}\ast {\widetilde {C}}_{t},\end{eqnarray*}

(6) \begin{eqnarray*}& & {h}_{t}={o}_{t}\ast tanh({C}_{t}).\end{eqnarray*}

We use *T* LSTM cells to form an encoder layer, and the *k*th layer is represented as *Le*^(*k*)^(⋅). The input of the first layer is *x*, and the input of each other layers are the output of previous layers. The output of each layer are the hidden states of LSTM cells in the corresponding layer. Thus, the encoder can be written as follows:

(7) \begin{eqnarray*}he{n}^{(k)}= \left\{ \begin{array}{@{}ll@{}} \displaystyle L{e}^{(k)}(x),\hspace*{10.00002pt}&\displaystyle k=1\\ \displaystyle L{e}^{(k)}(he{n}^{(k-1)}),\hspace*{10.00002pt}&\displaystyle k\gt 1 \end{array} \right. \end{eqnarray*}

where *hen*^(*k*)^ represents the hidden states of LSTM cells corresponding to the *k*th layer. If the hidden state of each LSTM cell is of size *M*, *hen*^(*k*)^ is of size *T* × *M*. We define *K* as the total number of encoding layers.

### Attention module

In this part, we integrate the encoder output with an attention module (Luong, Pham & Manning, 2015; Yang et al., 2016) so that the decoders can focus on important hidden patterns.

To build the module, we first perform a fully connected transformation for the encoder output and get a transformed state matrix $\bar {h}$:

(8) \begin{eqnarray*}\bar {h}=FCN(he{n}^{(K)}),\end{eqnarray*}

where *FCN*(*he*) represents the fully connected layer over hidden states, and the transformed state matrix $\bar {h}$ is a column vector of length *T* × *M*. We use *hl* to represent the last row of *hen*^(*K*)^. We obtain the attention score using $score(hl,{\bar {h}}_{t:})$ which is simply a dot product of two vectors. After normalization with a softmax function, we can obtain attention weight vector *aw* of length *T*, in which each element is defined as follows:

(9) \begin{eqnarray*}a{w}_{t}= \frac{\exp \nolimits (score(hl,{\bar {h}}_{t:}))}{\sum _{{t}^{{^{\prime}}}}\exp \nolimits (score(hl,{\bar {h}}_{{t}^{{^{\prime}}}:}))} ,\end{eqnarray*}

where ${\bar {h}}_{t:}$ is *t*th row of transformed state matrix $\bar {h}$.

Finally, we multiply the attention weight *aw* with $\bar {h}$ to obtain the attention vector:

(10) \begin{eqnarray*}av=a{w}^{T}\bar {h},\end{eqnarray*}

where *av* is a vector of length *M*.

The attention vector *av* is concatenated with *hl*, and fed into another fully connected layer to produce the final hidden pattern:

(11) \begin{eqnarray*}ha=FCN([av,hl]),\end{eqnarray*}

where *ha* is the attention output of length 2*M*.

#### LSTM decoders for trajectory reconstruction and prediction/imputation

A traditional auto-encoder model can be used for unsupervised learning and identify hidden patterns for trajectory series. In this work, we use a dual-decoder model to make it possible for supervised learning while utilizing the hidden patterns.

We use *Ld*^(*k*,1)^ and *Ld*^(*k*,2)^ to represent the *k*th LSTM layers of the first and the second decoders, respectively. Corresponding to the encoder in Eq. (7), the structure of the two decoders are as follows:

(12) \begin{eqnarray*}h{d}^{(k,1)}= \left\{ \begin{array}{@{}ll@{}} \displaystyle L{d}^{(k,1)}(ha),\hspace*{10.00002pt}&\displaystyle k=1\\ \displaystyle L{d}^{(k,1)}(h{d}^{(k,1)}),\hspace*{10.00002pt}&\displaystyle k\gt 1, \end{array} \right. ,\end{eqnarray*}

(13) \begin{eqnarray*}h{d}^{(k,2)}= \left\{ \begin{array}{@{}ll@{}} \displaystyle L{d}^{(k,2)}(ha),\hspace*{10.00002pt}&\displaystyle k=1\\ \displaystyle L{d}^{(k,2)}(h{d}^{(k,2)}),\hspace*{10.00002pt}&\displaystyle k\gt 1, \end{array} \right. ,\end{eqnarray*}

where *hd*^(*k*,1)^ represents the hidden states of LSTM cells corresponding to the *k*th layer of the first decoder, and *hd*^(*k*,2)^ represents that of the second decoder. The first decoder is used for reconstruction as usual so that it can help encoder to extract meaningful patterns from trajectories. Based on these patterns, the second decoder is for supervised learning, namely, predicting or imputation for the model input.

We use the outputs of the last layers of two decoders to compute the model outputs, and thus, if there are *K* decoder layers, we have

(14) \begin{eqnarray*}\widehat{x}=FCN(h{d}^{(K,2)}),\end{eqnarray*}

(15) \begin{eqnarray*}\widehat{y}=FCN(h{d}^{(K,2)}).\end{eqnarray*}

where $\widehat{x}$ is the reconstruction for the input data, and $\widehat{y}$ is the prediction or imputation result.

### Loss function

We choose the mean square error to construct the loss function for the whole framework. The loss function can compute the reconstruction error and the prediction or imputation error. In the specific task of our trajectory analysis, if *y* is the target label for input sequence *x*, with reconstruction sequence $\widehat{x}$ and the prediction or imputation output $\widehat{y}$, the objective of this model is to minimize the loss function:

(16) \begin{eqnarray*}\min \nolimits L=\sum _{j=1}^{n}[({x}^{(j)}-{\widehat{x}}^{(j)})^{2}+({y}^{(j)}-{\widehat{y}}^{(j)})^{2}],\end{eqnarray*}

where *n* is the number of trajectory segments and *j* represents the *j*th segment for input.

To train the model, we need to minimize the loss. Adam optimizer (Kingma & Ba, 2014) is widely used for many deep learning models, so we also use it to minimize the loss function.

## Experiments

### Dataset

We use a data set that includes trajectories of 489,391 h from 111 southern elephant seals and their positions obtained from Argos platform transmitter terminals. All procedures to obtain the data were approved by the respective ethics committees and licensing bodies including, the Australian Antarctic Animal Ethics Committee (ASAC 2265, AAS 2794, AAS 4329), the Tasmanian Parks and Wildlife Service, the University of California, Santa Cruz, and the Programa Antártico Brasileiro. This procedure is carried out in accordance with current guidelines and regulations.

### Data preprocessing

**Figure 2:**
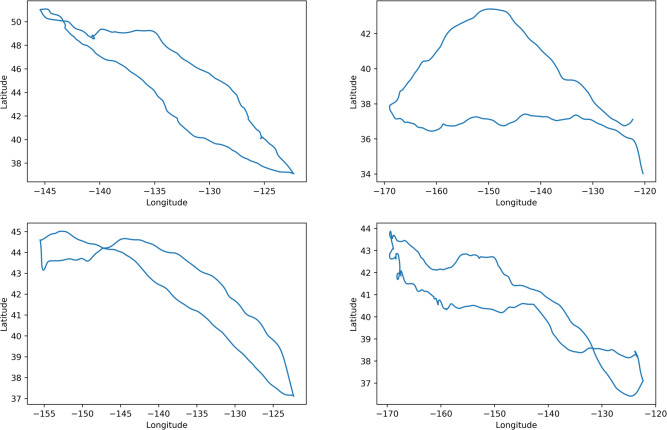
Example trajectories of some elephant seals.

Our method can take position information, including longitudes and latitudes, into account obtained from animal trajectories. However, although the data set is quite large, animals usually appear at different positions. Figure 2 shows such scenarios with four Antarctic elephant seals.

To solve this issue, we feed our algorithm with distances and angles information extracted from trajectories for ease of learning. We use *P*_*t*_ to denote the position in longitude and latitude at time *t*. We use *d*_*t*_ to denote the distance traveled during the period *t* between two data collections. We also use *θ*_*t*_ to indicate the direction of movement. Therefore, with longitude and latitude information, *d*_*t*_ represents the great-circle distance between *P*_*t*_ and *P*_*t*+1_ calculated by haversine equation, and *θ*_*t*_ represents the azimuth angle of the direction from *P*_*t*−1_*P*_*t*_ to *P*_*t*_*P*_*t*+1_. The input *x* of our model includes following features (*d*_*t*_cos*θ*_*t*_, *d*_*t*_sin*θ*_*t*_, *θ*_*t*_), and the output of our model is (*d*_*t*_cos*θ*_*t*_, *d*_*t*_sin*θ*_*t*_).

We also slice the trajectory data into segments with a sliding window. Each segment has a certain number of consecutive data points. The number of data points in each segment would vary depending on the experiment.

### Experiment design

In our experiments, we consider three cases to prepare the training and testing data:

1. One seal: in this case, each experiment is carried out within one seal’s data. We use half of the trajectory data for training and the other half for testing. The first half of a seal trajectory is used as a training set, and the second half is as a testing set.
2. Five seals: in this case, each experiment is carried out with four seals for training and one seal for testing. Testing seals are not included in the training set.
3. All seals: in this case, we first extract trajectory segments of all the seals and then randomly shuffle these segments. In the experiment, we use the first half of the shuffled segments for training and the other half for testing.

To evaluate the efficiency of our model with and without attention (LSTM-AE-ATDD and LSTM-AE-DD), we choose three other methods for comparison. These models have also been widely used in trajectory prediction and imputation tasks. The first one is a widely used but simple LSTM model having one hidden layer of one hundred neurons for analyzing sequence data.

The second method is a densely connected artificial neural network (ANN), in which there is a hidden layer with one hundred neurons. The third one is a random forest method with two hundred decision trees. It is an ensemble method that proved to be effective for time series regression. For simplicity, we choose the single-layer encoder and decoders in our approach.

For evaluation, we select two metrics, Mean Absolute Error (MAE) and Root Mean Square Error (RMSE), for the model output *d*_*t*_cos*θ*_*t*_ and *d*_*t*_sin*θ*_*t*_ when comparing with the ground-truth.

### Data prediction

In this part, we consider the application of data prediction. Given an input trajectory, our model generates location information for time steps following the input sequence. We evaluate the impact of differences in segment length for training and testing. The notations are shown as in Table 1. For example, *T*_7_*P*_1_ means that we use the first seven time steps of a segment as input, and the model produces results for the eighth time step. We also compare our approach with other methods to evaluate its performance. For evaluation, we also select MAE and RMSE for the model output *d*_*t*_*cosθ*_*t*_ and *d*_*t*_*sinθ*_*t*_ when comparing with the ground-truth.

**Table 1:** Notations for differences in segment length in data prediction.

**Subcases**	**Length for input**	**Length for output**
*T*_7_ *P*_1_	7	1
*T*_7_ *P*_4_	7	4
*T*_12_ *P*_7_	12	7

#### Case 1: One Seal

**Figure 3:**
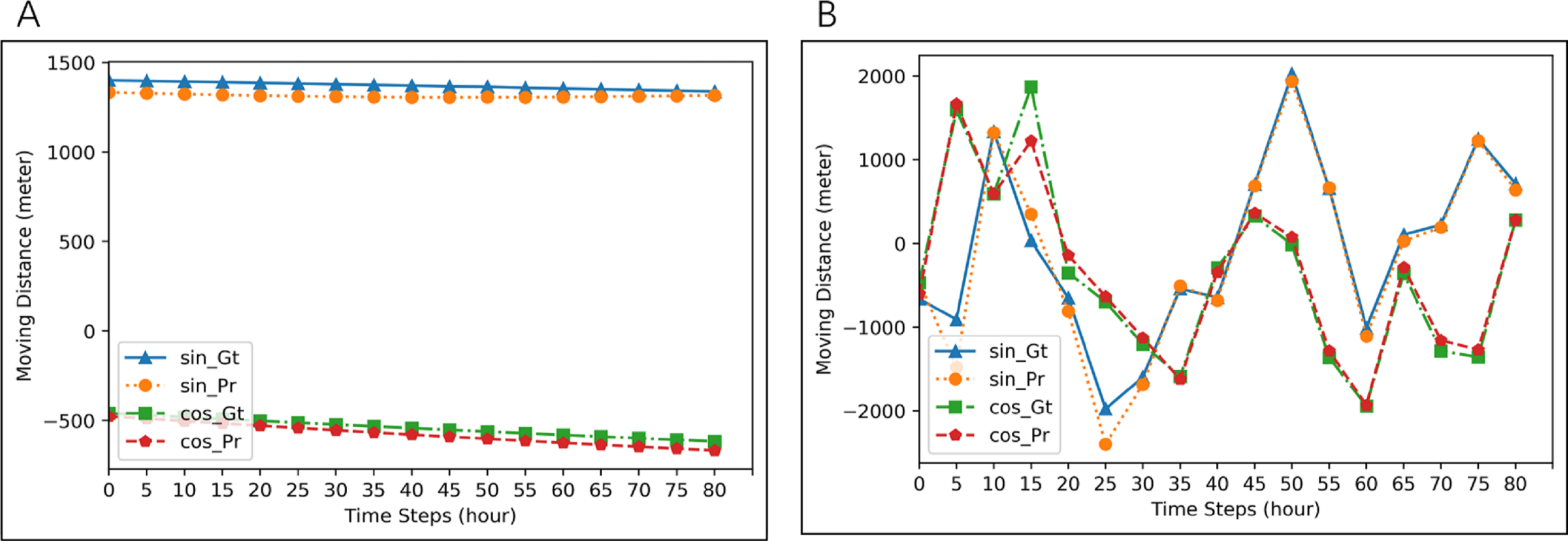
Examples of prediction for two different seals. Blue lines (ground-truth) and orange line (prediction) are for *d*_*t*_cos*θt*. Red lines (ground-truth) and green lines (prediction) are for *d*_*t*_sin*θt*. MAE of prediction in (A) is 1.489, and that in (B) is 688.8166.

In this case, for each experiment, we use trajectory segments from one seal for training and testing. We use 80% of the data for training and the remaining 20% for testing. We carry out one experiment for each seal and then calculate the average performance for all the experiments. Results are shown in Tables 2 and 3. Comparing with other methods in Table 2, the average MAE of LSTM-AE-ATDD is 19.47% less than that of LSTM, 71.81% less than that of ANN, and 51.49% less than that of Random forests. From Table 3, we can find that the average RMSE of LSTM-AE-ATDD is 22.57% less than that of LSTM, 62.87% less than that of ANN, and 46.40% less than that of Random forests. These results demonstrate the effectiveness of our model. Example predicting results by our approach are shown in Fig. 3.

**Table 2:** MAE for prediction with One Seal case.

	**LSTM-AE-ATDD**	**LSTM-AE-DD**	**LSTM**	**ANN**	**Random forests**
*T*_7_ *P*_1_	**237.319**	250.440	269.904	1163.260	583.062
*T*_7_ *P*_4_	**323.735**	349.914	375.148	1162.241	657.521
*T*_12_ *P*_7_	**420.102**	462.545	473.786	1152.049	780.859

**Table 3:** RMSE for prediction with One Seal case.

****	**LSTM-AE-ATDD**	**LSTM-AE-DD**	**LSTM**	**ANN**	**Random forests**
*T*_7_ *P*_1_	**387.995**	408.143	484.409	1448.455	860.921
*T*_7_ *P*_4_	**503.205**	806.414	701.441	1514.741	937.436
*T*_12_ *P*_7_	**657.439**	753.896	813.486	1504.378	1089.780

#### Case 2: Five Seals

**Figure 4:**
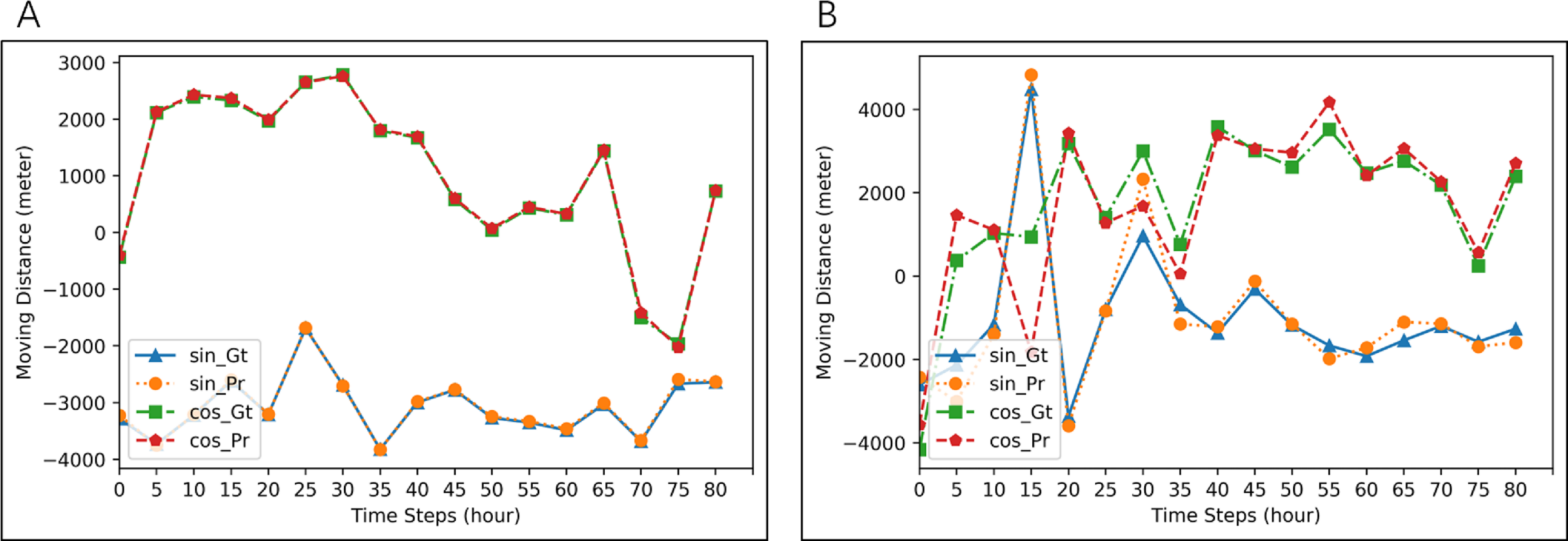
Examples of prediction for two groups of five seals. Blue lines (ground-truth) and orange lines (predicted values) are for *d*_*t*_cos*θt*. Red lines (ground-truth) and Green (prediction) are for *d*_*t*_sin*θt*. MAE of prediction in (A) is 2.7896, and that in (B) is 666.761.

In this case, seals data are randomly divided into multiple groups, with each group includes trajectory segments from five seals. We use one group of seals for each experiment and choose segments from four seals in the group as training data and segments from the other seal in the group as testing data. We carry out experiments for all the groups and calculated the average performance. Results are shown in Tables 4 and 5. Comparing with other methods in Table 4, the average MAE of LSTM-AE-ATDD is 11.88% less than that of LSTM, 75.90% less than that of ANN, and 23.31% less than that of Random forests. From Table 5, we can find that the average RMSE of LSTM-AE-ATDD is 22.13% less than that of LSTM, 72.22% less than that of ANN, and 20.54% less than that of Random forests. These results demonstrate the effectiveness of our model. Example segments are shown in Fig. 4.

**Table 4:** MAE for prediction with Five Seals case.

****	**LSTM-AE-ATDD**	**LSTM-AE-DD**	**LSTM**	**ANN**	**Random forests**
*T*_7_ *P*_1_	**168.918**	187.926	198.6611	946.406	239.127
*T*_7_ *P*_4_	**249.005**	270.423	277.894	1021.269	316.594
*T*_12_ *P*_7_	**310.115**	343.232	350.289	1050.041	393.695

**Table 5:** RMSE for prediction with Five Seals case.

****	**LSTM-AE-ATDD**	**LSTM-AE-DD**	**LSTM**	**ANN**	**Random forests**
*T*_7_ *P*_1_	**277.966**	348.854	347.808	1375.048	389.258
*T*_7_ *P*_4_	**408.962**	460.440	548.086	1458.451	495.996
*T*_12_ *P*_7_	**522.188**	605.666	655.394	1511.277	635.0766

**Table 6:** MAE for prediction with All Seals case.

****	**LSTM-AE-ATDD**	**LSTM-AE-DD**	**LSTM**	**ANN**	**Random forests**
*T*_7_ *P*_1_	**1362.880**	1510.908	1627.762	1973.107	1823.014
*T*_7_ *P*_4_	**1350.552**	1552.378	1587.598	1957.022	1846.123
*T*_12_ *P*_7_	**1544.437**	1562.605	1569.930	1947.542	1860.650

**Table 7:** RMSE for prediction with All Seals case.

****	**LSTM-AE-ATDD**	**LSTM-AE-DD**	**LSTM**	**ANN**	**Random forests**
*T*_7_ *P*_1_	**1910.567**	2175.505	2254.435	2411.910	2289.266
*T*_7_ *P*_4_	**1802.803**	2061.795	2124.295	2434.975	2311.551
*T*_12_ *P*_7_	**2039.647**	2075.911	2083.032	2398.189	2355.768

#### Case 3: All Seals

**Figure 5:**
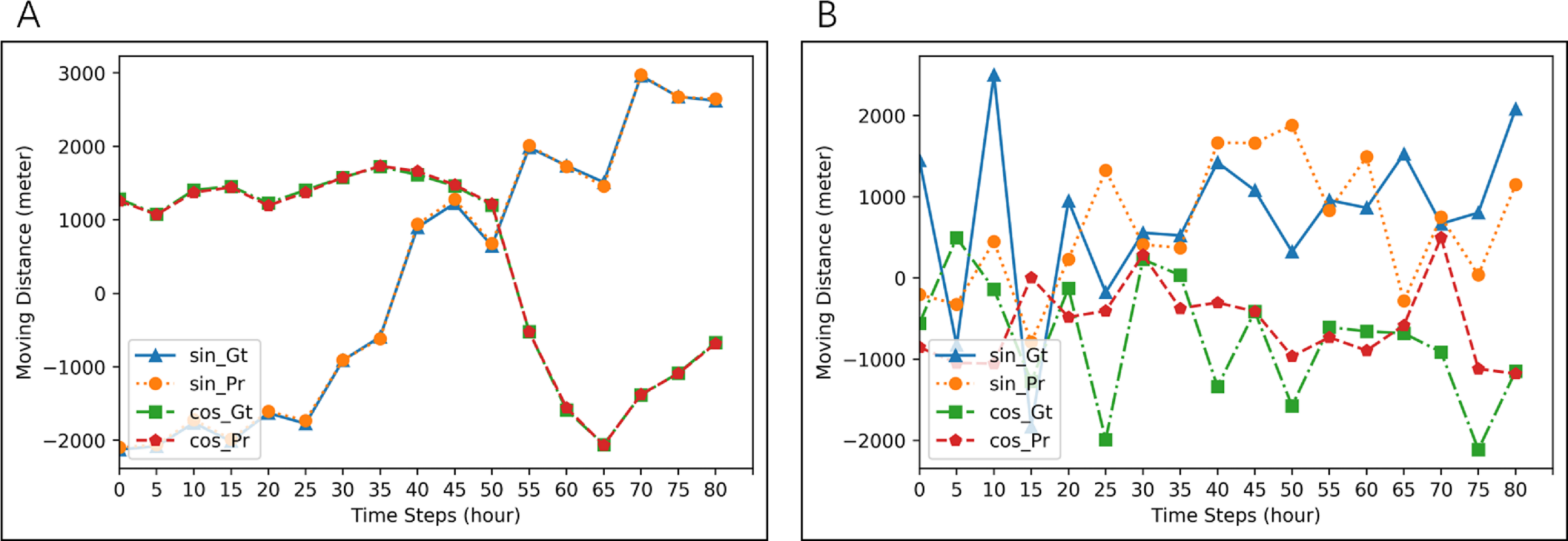
Examples of prediction for all seals. Blue lines (ground-truth) and orange lines (prediction) are for *d*_*t*_cos*θt*. Red lines (ground-truth) and green lines (prediction) are for *d*_*t*_sin*θt*. MAE of prediction in (A) is 29.6425, and that in (B) is 723.7696.

In this case, we use all the segments from all the seals in the experiment. We randomly choose half of the segments for training and the other half for testing. Results are shown in Tables 6 and 7. Comparing with other methods in Table 6, the average MAE of LSTM-AE-ATDD is 11.02% less than that of LSTM, 27.58% less than that of ANN, and 23.02% less than that of Random forests. From Table 7, we can find that the average RMSE of LSTM-AE-ATDD is 10.99% less than that of LSTM, 20.60% less than that of ANN, and 17.31% less than that of Random forests. These results demonstrate the effectiveness of our model. Example segments are shown in Fig. 5.

**Table 8:** Notations for differences in segment length in data imputation.

**Subcases**	**Length for input**	**Length for output**
*T*_1_ *P*_1_	1	1
*T*_7_ *P*_7_	7	7
*T*_14_ *P*_14_	14	14

**Table 9:** MAE for imputation with One Seal case.

****	**LSTM-AE-ATDD**	**LSTM-AE-DD**	**LSTM**	**ANN**	**Random forests**
*T*_1_ *P*_1_	281.664	**208.968**	240.295	814.662	505.864
*T*_7_ *P*_7_	**236.992**	236.997	257.502	997.410	706.942
*T*_14_ *P*_14_	**243.321**	286.144	301.394	946.278	879.690

**Table 10:** RMSE for imputation with One Seal case.

****	**LSTM-AE-ATDD**	**LSTM-AE-DD**	**LSTM**	**ANN**	**Random forests**
*T*_1_ *P*_1_	507.709	**389.9149**	441.229	1136.288	799.169
*T*_7_ *P*_7_	**405.793**	420.320	457.572	1415.251	1001.373
*T*_14_ *P*_14_	**402.579**	499.870	537.589	1286.640	1177.041

**Figure 6:**
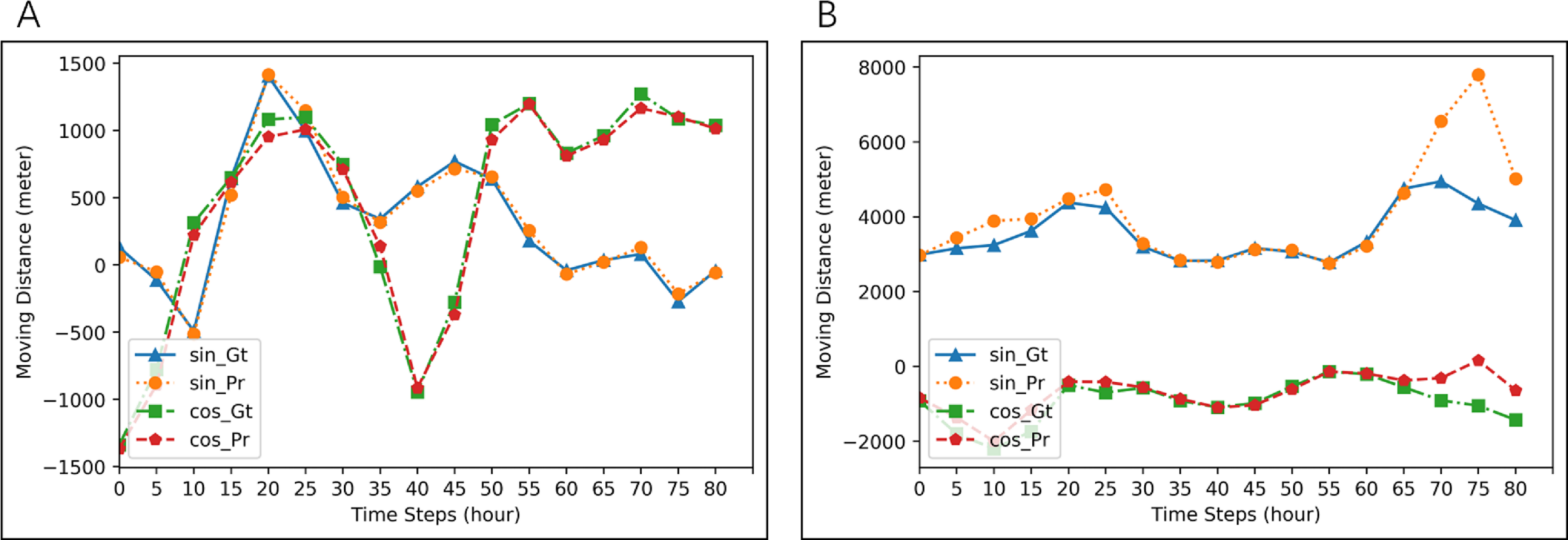
Examples of imputation for two different seals. Blue lines (ground-truth) and orange lines (imputation) are for *d*_*t*_cos*θt*. Red lines (ground-truth) and green line (imputation) are for *d*_*t*_sin*θt*. MAE of imputation in (A) is 3.2423, and that in (B) is 652.1680.

### Data imputation

In this part, we consider the application of data imputation. It is to generate missing data points for given sequences. We carry out a comprehensive evaluation with three different cases. The output segments are the same as the input length as the item in Table 8. For example, notation *T*_7_*P*_7_ means that we use a total of fourteen time steps, with seven steps corresponding to time {1, 3, 5, 7, 9, 11, 13} for input, and the other seven steps corresponding to time {2, 4, 6, 8, 10, 12, 14} as output. We also compare our approach with other methods to evaluate its performance. For evaluation, we also select MAE and RMSE for the model output *d*_*t*_*cosθ*_*t*_ and *d*_*t*_*sinθ*_*t*_ when comparing with the ground-truth.

#### Case 1: One Seal

In this case, for each experiment, we use one seal for training and testing. The length of the sequence is set to be 1, 7 and 14 respectively. For one seal, we use 80% of the segments for training and the remaining 20% for testing. We carry out such experiments for all the seals and calculated the average performance. Results are shown in Tables 9 and 10. Comparing with other methods, both of our approaches are effective, but LSTM-AE-ATDD is not as good as LSTM-AE-DD for *T*_1_*P*_1_, which is reasonable because the input segment with length one is too short for attention mechanism to work. From Table 9, we can find that the average MAE of LSTM-AE-ATDD is 8.52% less than that of LSTM, 73.52% less than that of ANN, and 65.07% less than that of Random forests. From Table 10, we can find that the average RMSE of LSTM-AE-ATDD is 8.85% less than that of LSTM, 65.91% less than that of ANN, and 56.06% less than that of Random forests. Example imputation results are shown in Fig. 6.

#### Case 2: Five Seals

**Figure 7:**
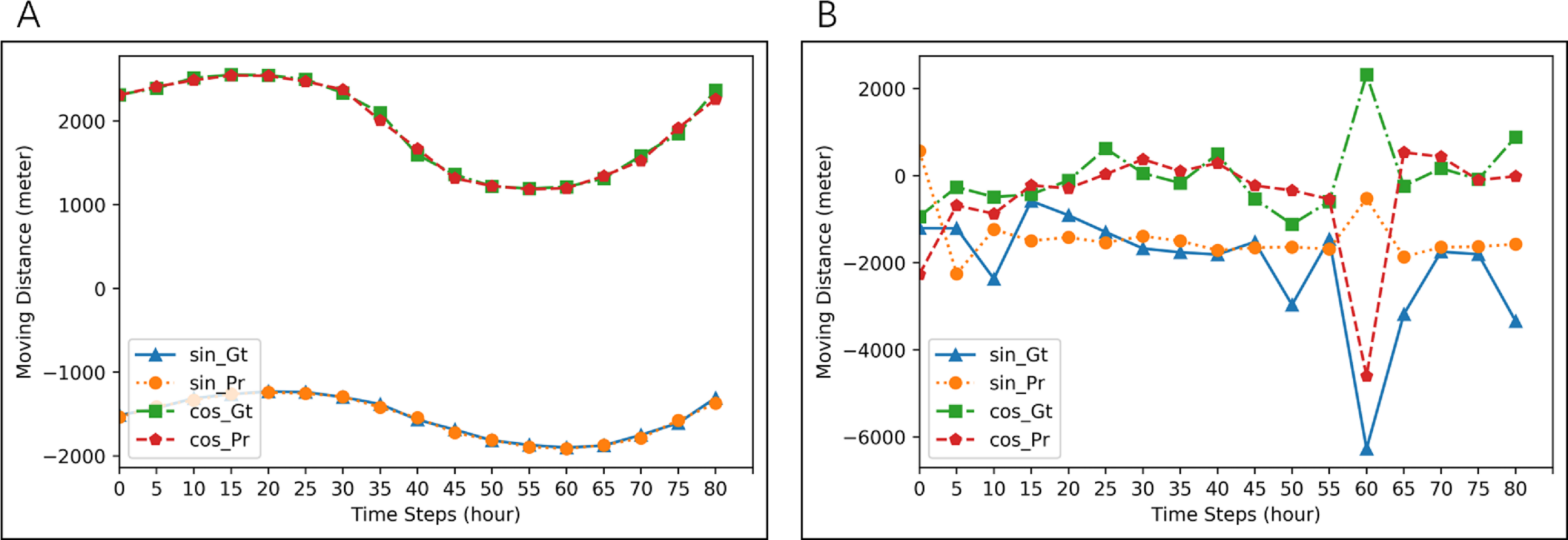
Examples of imputation results for two groups of five seals. Blue lines (ground-truth) and orange lines (imputation) are for *d*_*t*_cos*θt*. Red lines (ground-truth) and green lines (imputation) are for *d*_*t*_sin*θt*. MAE of imputation in (A) is 16.9761, and that in (B) is 689.0033.

In this case, seals data are randomly divided into multiple groups, with each group includes trajectory segments from five seals. We use one group of seals for each experiment and choose segments from four seals in the group as training data and segments from the other seal in the group as testing data. We carry out experiments on all the groups and calculated the average performance. Results are shown in Tables 11 and 12. Comparisons with other methods prove the effectiveness of our approach, and similar as before, LSTM-AE-ATDD is not as good as LSTM-AE-DD for *T*_1_*P*_1_ because the input segment with length one is too short for attention mechanism to work. From Table 11, we can find that the average MAE of LSTM-AE-ATDD is 40.33% less than that of LSTM, 75.34% less than that of ANN, and 31.49% less than that of Random forests. From Table 12, we can find that the average RMSE of LSTM-AE-ATDD is 25.89% less than that of LSTM, 70.49% less than that of ANN, and 24.77% less than that of Random forests. Example segments are shown in Fig. 7.

**Table 11:** MAE for imputation with Five Seals case.

****	**LSTM-AE-ATDD**	**LSTM-AE-DD**	**LSTM**	**ANN**	**Random forests**
*T*_1_ *P*_1_	190.748	**149.283**	151.353	918.250	260.823
*T*_7_ *P*_7_	**159.890**	176.549	192.877	934.221	271.680
*T*_14_ *P*_14_	**345.679**	450.622	820.294	962.841	482.425

**Table 12:** RMSE for imputation with Five Seals case.

****	**LSTM-AE-ATDD**	**LSTM-AE-DD**	**LSTM**	**ANN**	**Random forests**
*T*_1_ *P*_1_	366.756	**291.972**	300.275	1385.303	476.676
*T*_7_ *P*_7_	**308.363**	336.048	365.393	1403.790	482.591
*T*_14_ *P*_14_	**571.478**	897.523	1015.749	1431.606	697.706

#### Case 3: All Seals

**Figure 8:**
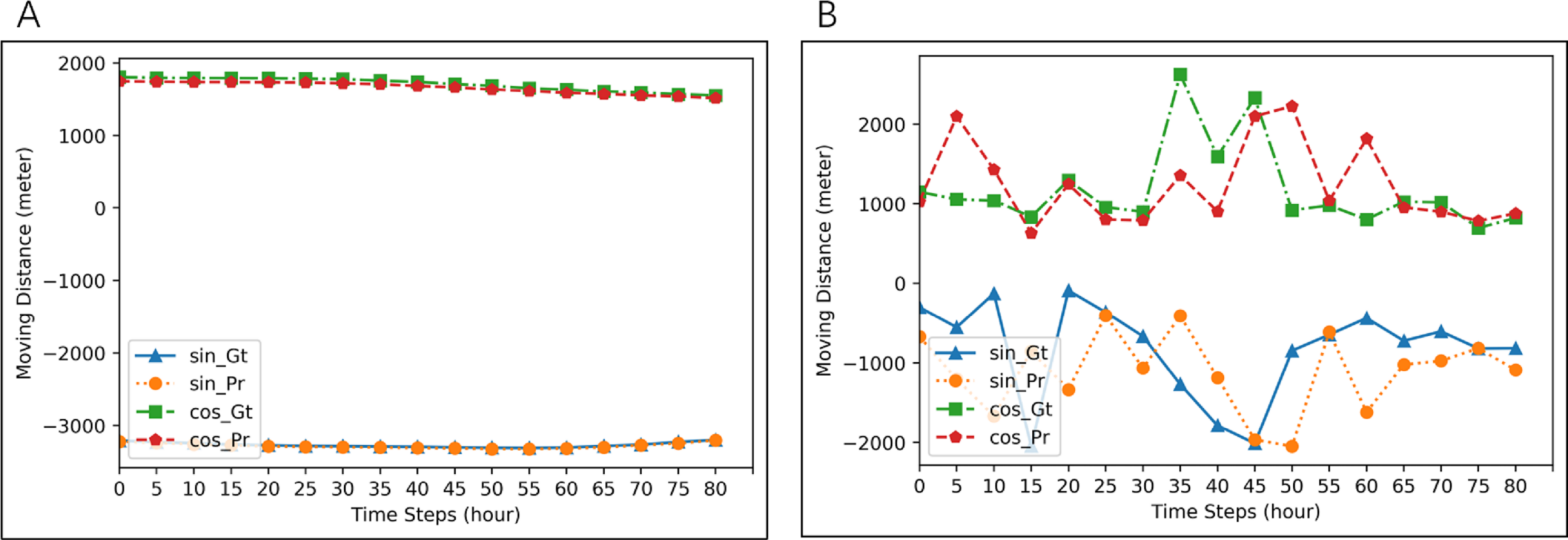
Examples of imputation results for all seals. Blue lines (ground-truth) and orange lines (imputation) are for *d*_*t*_cos*θt*. Red lines (ground-truth) and green lines (imputation) are for *d*_*t*_sin*θt*. MAE of imputation in (A) is 10.8995, and that in (B) is 672.6100.

In this case, we use all the segments from all the seals in the experiment. We randomly choose half of the segments for training and the other half for testing. Results are shown in Tables 13 and 14. Comparisons with other methods prove the effectiveness of our approach, especially imputation for long sequences. In this experiment, LSTM-AE-DD is always slightly better than LSTM-AE-ATDD, probably because behaviors of seals may diverge, making it difficult for the attention mechanism to catch patterns of all the seals properly. From Table 13, we can find that the average MAE of LSTM-AE-ATDD is 47.70% less than that of LSTM, 79.06% less than that of ANN, and 78.55% less than that of Random forests. From Table 14, we can find that the average RMSE of LSTM-AE-ATDD is 28.03% less than that of LSTM, 52.78% less than that of ANN, and 52.68% less than that of Random forests. Example segments are shown in Fig. 8.

**Table 13:** MAE for imputation with All Seals case.

****	**LSTM-AE-ATDD**	**LSTM-AE-DD**	**LSTM**	**ANN**	**Random forests**
*T*_1_ *P*_1_	203.770	**200.627**	201.826	903.516	894.184
*T*_7_ *P*_7_	209.046	**200.736**	207.016	984.989	941.229
*T*_14_ *P*_14_	206.405	**204.781**	747.974	997.401	981.847

**Table 14:** RMSE for imputation with All Seals case.

****	**LSTM-AE-ATDD**	**LSTM-AE-DD**	**LSTM**	**ANN**	**Random forests**
*T*_1_ *P*_1_	758.635	**736.740**	751.158	1503.748	1500.653
*T*_7_ *P*_7_	752.292	**731.636**	774.091	1581.619	1579.469
*T*_14_ *P*_14_	739.197	**718.632**	1511.687	1543.229	1538.450

## Conclusions

Trajectory prediction and imputation are essential in analyzing trajectory data. In this work, we propose an approach utilizing auto-encoders and attention modules to extract important hidden patterns and then use an additional decoder for estimation. This approach can overcome the drawback raised with pure prediction or imputation networks. The proposed attention module for the hidden patterns can further select critical patterns for decoders, and thus, it improves prediction and imputation results. In the experiments, our model performs better than others, which proves the effectiveness of our approach. This method can meet a wide range of applications for biologists and ecologists.

## Supplemental Information

10.7717/peerj-cs.656/supp-1 Supplemental Information 1 Code for the analysis

## Additional Information and Declarations

### Data Availability

The following information was supplied regarding data availability: The data is available in the Supplemental File and at the following: Roquet, F., Guinet, C., & Hindell, Southern Elephant Seal CTD tag data 2004 - 2013, M. A. NERC Data Centres http://dx.doi.org/10/srk (2014) British Oceanographic Data Centre (2017). Southern Elephant Seal CTD tag data 2004–2013. https://data-search.nerc.ac.uk:443/geonetwork/srv/api/records/6aac2d279f6e58f9bd66ca7c44173ae6
https://www.bodc.ac.uk/resources/inventories/edmed/report/6072/.
